# Comparison of sexual function in women with endometrial cancer and their partners following surgery versus surgery additional brachytherapy: a cross-sectional study from a tertiary care center

**DOI:** 10.3389/fmed.2025.1697957

**Published:** 2025-11-28

**Authors:** Gazi Güner, Can Tercan, Emrah Dagdeviren, Ayşe Bacaksız, Erdem Kiray, Sure Nur Erdoğmuş, Yahya Özgün Öner, Sinem Özşahin Kılıç, Zeliha Zeynep Satılmışoğlu, Çisem Ertok, Ergün Tercan, Figen Efe Çamili, Nazlı Aylin Vural, Ayben Yentek Balkanay, İlkbal Temel Yüksel, Selçuk Erkılınç

**Affiliations:** 1 Department of Gynecologic Oncology Surgery, Başakşehir Çam and Sakura City Hospital, Istanbul, Türkiye; 2 Department of Obstetrics and Gynecology, Başakşehir Çam and Sakura City Hospital, Istanbul, Türkiye; 3 Department of Gynecologic Oncology Surgery, Sivas Numune Hospital, Sivas, Türkiye; 4 Department of Gynecologic Oncology, Bursa Yüksek İhtisas Training and Research Hospital, Bursa, Türkiye; 5 Department of Pathology, Manisa Merkezefendi State Hospital, Manisa, Türkiye; 6 Department of Obstetrics and Gynecology, Faculty of Medicine, Balıkesir University, Balıkesir, Türkiye; 7 Department of Gynecologic Oncology Surgery, Yozgat City Hospital, Yozgat, Türkiye; 8 Department of Radiation Oncology, Başakşehir Çam and Sakura City Hospital, Istanbul, Türkiye; 9 Department of Gynecologic Oncology Surgery, Buca Seyfi Demirsoy Training and Research Hospital, İzmir Democracy University, Izmir, Türkiye

**Keywords:** endometrial cancer, brachytherapy, sexual function, female sexual function index (FSFI), new sexual satisfaction scale (NSSS), partner satisfaction, gynecologic oncology, quality of life

## Abstract

**Background:**

This study aimed to compare female sexual function and male partner sexual satisfaction between endometrial cancer patients treated with surgery alone and those receiving additional brachytherapy.

**Methods:**

Sixty-nine patients were included. Group 1 (*n* = 34) received adjuvant brachytherapy after surgery; Group 2 (*n* = 35) underwent surgery only. Participants completed a structured questionnaire including socio-demographic and clinical data, along with validated instruments: the Female Sexual Function Index and the New Sexual Satisfaction Scale for partners.

**Results:**

The mean age was significantly higher in the brachytherapy group (61.71 ± 7.88 vs. 56.54 ± 8.74 years; *p* = 0.012). Gravidity (*p* = 0.029) and parity (*p* = 0.013) were also higher in this group, while body mass index was similar (*p* = 0.118). Female sexual function index scores [2 (2–20.3) vs. 2 (2–19.6); *p* = 0.459] and new sexual satisfaction scale scores [20 (20–83) vs. 20 (20–90); *p* = 0.492] showed no significant differences. Female sexual function index subdomain scores were also comparable. Tumor grade and stage significantly differed between groups, as did surgical approach and lymphadenectomy rates.

**Conclusion:**

Sexual function was negatively affected in all endometrial cancer patients, with a more pronounced impact among those receiving brachytherapy and their partners. These findings highlight the need to consider sexual health in treatment planning and to implement supportive interventions such as psychosexual counseling, particularly for patients undergoing adjuvant radiotherapy.

## Introduction

Endometrial cancer is the most common cancer of the female reproductive system and its incidence is increasing due to the aging and the obesity epidemic. About 85% are usually diagnosed in the early stage and has a excellent prognosis (1). Endometrial cancer represents one of the most prevalent gynecologic malignancies in developed nations, accounting for nearly 30–40% of all uterine cancers, with a steadily rising global incidence. According to recent epidemiologic data, the disease predominantly affects postmenopausal women, with the median age at diagnosis ranging from 60 to 65 years, although cases among younger women have gradually increased due to obesity and metabolic syndrome related risk factors (2). Advances in molecular characterization particularly the integration of genomic and transcriptomic profiling have refined diagnostic accuracy and risk stratification, enabling the adoption of more personalized and risk-adapted treatment approaches (3). Moreover, novel bioengineering and imaging innovations, including high resolution multiparametric MRI and AI-assisted modeling, have enhanced the precision of assessing tumor invasion, lymphovascular spread, and treatment response, thereby improving clinical decision-making and survivorship outcomes (4). Adjuvant radiotherapy is applied to reduce the risk of recurrence in endometrial cancer patients. Brachitherapy targets the first third of the vagina and the vaginal cuff, applied to a depth of 5 mm. Depending on pathology results, the dose depth may be increased toward the paravaginal region or parametrium (5). Organ-sparing dose limitations are based on the limitations defined for cervical cancer treatments. Bladder, rectum and bowel toxicities have been identified during the development of dose optimization in general, and the vagina has been limitedly included among the organs at risk in radiotherapy planning (6–9). Vaginal dryness, vaginal stenosis and loss of elasticity, fibrosis, inflammation, mucosal damage and ulceration are among the negative side effects of brachytherapy on the vagina. These physical effects can make sexual intercourse unpleasant or painful. Also, changes in the function and structure of the genitals can lead to feelings of inadequacy, emotional detachment and guilt (10). In addition to the physical changes, these psychosexual effects result in anxiety, depression and aversion. More than 40% of women want to receive sexual counselling after treatment, yet these side effects are ignored and under-treated in patient management (11). In our study, we aimed to evaluate the impact of the surgical procedure and brachytherapy used in the treatment of endometrial cancer on the sexual life of couples.

To the best of our knowledge, this is the first study to simultaneously evaluate sexual function in endometrial cancer patients and their partners.

## Materials and methods

### Study design

This study was designed as a retrospective analysis of patients diagnosed with endometrial cancer, followed by a prospective, observational, and comparative survey conducted among women who underwent surgery alone or surgery with additional brachytherapy, as well as their partners. The Female Sexual Function Index (FSFI) and the New Sexual Satisfaction Scale (NSSS) were used to assess female and partner sexual function, respectively. This study was designed as a retrospective patient chart review combined with a prospective survey analysis. Patients diagnosed with endometrial cancer were retrospectively identified from institutional medical records, while the assessment of sexual function was conducted prospectively through validated questionnaires. The study aimed to evaluate the sexual function of women with endometrial cancer who received or did not receive radiotherapy, and to assess their partners’ sexual satisfaction. The Female Sexual Function Index (FSFI) and the New Sexual Satisfaction Scale (NSSS) were used to assess female and partner sexual function, respectively. All the questionnaires were administered face to face.

### Participants

A total of 69 women diagnosed with endometrial cancer and their heterosexual partners were included in this study. All participants were treated at Başakşehir Çam and Sakura City Hospital between January 1, 2021, and March 31, 2024. Patients who had undergone primary surgery followed by adjuvant radiotherapy were assigned to the radiotherapy group, while those who underwent surgery alone were assigned to the surgery-only group. Their male partners were also included in the study. All participants provided written informed consent prior to enrollment. Exclusion criteria included being under 18 years of age, having chronic psychiatric disorders, being sexually inactive, or having a diagnosis other than endometrial carcinoma and having a psychiatric disorder.

**Figure 1:**
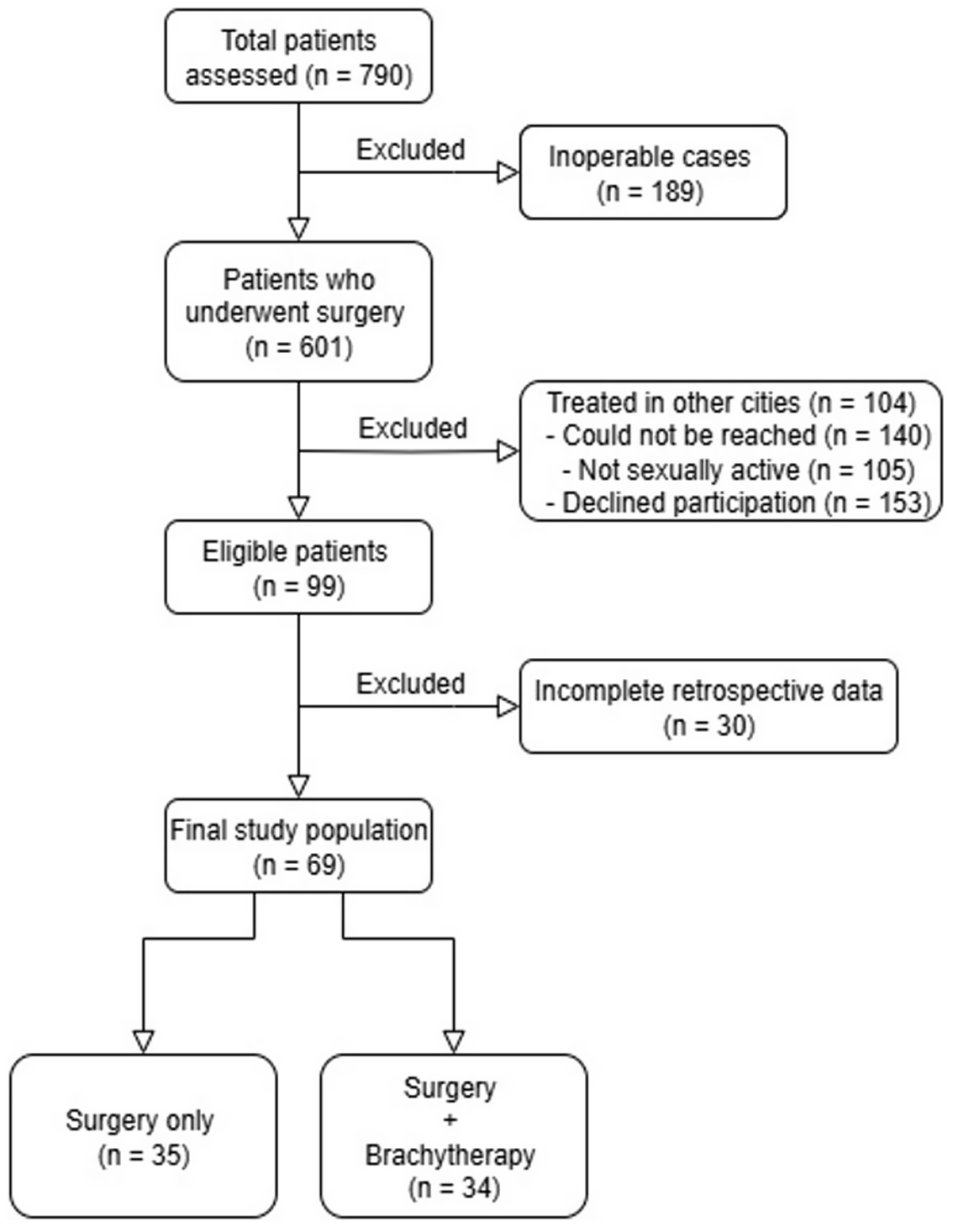
Patient selection diagram.

Sample size estimation was based on an effect size of Cohen’s d = 0.8, with 80% power and a significance level of *α* = 0.05, indicating that at least 64 participants per group (128 total) were required. To account for potential dropouts, a total of 70 patients (35 per group) and their partners (140 participants in total) were targeted. Between January 2021 and March 2024, 790 patients were diagnosed with endometrial cancer at our institution. Of these, 189 were inoperable, and 601 underwent surgery. Among operated patients, 104 continued follow-up in other provinces, 140 were unreachable, 105 were sexually inactive, and 153 declined participation for personal, cultural, or religious reasons. Of the remaining 99 eligible patients, 30 were excluded due to incomplete data, yielding a final cohort of 69 patients who met the inclusion criteria. Among these, 35 patients underwent surgery alone, while 34 patients received adjuvant brachytherapy following surgery. The present study compared these two groups in terms of demographic, clinical, and sexual function outcomes (Figure 1). Adjuvant radiotherapy decisions were made in accordance with the 2023 ESGO–ESTRO–ESP guidelines for the management of endometrial cancer (12). Since all patients were in early stage chemotherapy was not administered

### Data collection

Sociodemographic and clinical data were obtained using a structured Participant Information Form. Sexual function and satisfaction were assessed using validated instruments: The FSFI is a 19-item scale evaluating six domains of female sexual function (desire, arousal, lubrication, orgasm, satisfaction, and pain), with total scores ranging from 2 to 36; scores below 26.55 indicate dysfunction. Severity was classified as severe (2.0–7.2), moderate (7.3–14.4), mild to moderate (14.5–21.6), mild (21.7–28.1), and none (28.2–36.0) (13). The NSSS is a 20-item, 100-point scale assessing overall sexual satisfaction, including emotional and relational aspects. Higher scores indicate greater satisfaction. For male participants, satisfaction was categorized as low (≤40), moderate (41–60), or high (>60). As validated cutoff points for this scale have not been established in previous studies, cutoff values were defined by the authors according to the distribution characteristics of the present dataset.

### Statistical analysis

Data were analyzed using Jamovi 2.3.28 and SPSS 26. Normality was tested with Kolmogorov–Smirnov and Shapiro–Wilk tests, revealing non-normal distributions; thus, nonparametric tests were applied. Between-group comparisons used Mann–Whitney U or *t*-tests as appropriate, and correlations were assessed with Pearson analysis. Multivariate regression identified independent predictors of sexual dysfunction and satisfaction. A *p* < 0.05 was considered significant. Power analysis (*α* = 0.05, power = 0.80) indicated 62 required participants; the final cohort of 69 achieved a post-hoc power of 0.90.

### Ethical approval

This study was reviewed and approved by the Clinical Research Ethics Committee of Başakşehir Çam and Sakura City Hospital, Istanbul, Türkiye (Approval No: KAEK-11/16.10.2024.194). Official approval was granted on 16 December 2024.

All procedures were performed in accordance with the ethical standards of the institutional and national research committees and with the Declaration of Helsinki. Written informed consent was obtained from all participants.

## Results

A total of 69 patients were included in the final analysis, comprising 34 women who underwent surgery with adjuvant brachytherapy and 35 who underwent surgery alone.

The Female Sexual Function Index (FSFI) uses a Likert-type response format, with items scored on a scale ranging from 0 or 1 to 5, depending on the question. In the present study, the internal consistency of the FSFI was excellent, with a Cronbach’s alpha coefficient of 0.93, confirming the reliability of the instrument.

The internal consistency of the NSSS was excellent, with a Cronbach’s alpha coefficient of 0.81 confirming the reliability of the scale in our study population. The mean total NSSS score was 81, indicating a generally high level of sexual satisfaction among male partners.

### Patient characteristics (Age, BMI, education, obstetric history, vs.)

The brachytherapy group was older (61.7 ± 7.9 vs. 56.5 ± 8.7 years, *p* = 0.012) and had higher gravidity and parity (*p* < 0.05), while BMI was similar between groups. Other demographic and lifestyle characteristics, including education, contraceptive use, smoking, and chronic disease status, showed no significant differences (Table 1).

**Table 1:** Baseline demographic and obstetric characteristics by treatment group.

Variable		Brachytherapy	Surgery	*p*-value
Age (years)* (*n* = 34/*n* = 35)		61.71 ± 7.88	56.54 ± 8.74	**0.012**
BMI (kg/m^2^)* (*n* = 34/*n* = 35)		33.94 ± 6.14	31.61 ± 6.04	0.118
Gravidity** (*n* = 34/*n* = 35)		3.5 (0–9)	3 (0–5)	**0.029**
Parity** (*n* = 34/*n* = 35)		3 (0–6)	2 (0–5)	**0.013**
Education level*** (*n* = 34/*n* = 32)	No education	5 (15.2%)	5 (15.6%)	0.300
	Primary school	22 (66.7%)	14 (43.8%)	
	Middle school	2 (6.1%)	4 (12.5%)	
	High school	4 (12.1%)	8 (25.0%)	
	University	0 (0.0%)	1 (3.1%)	
Contraceptive Use*** (*n* = 33/*n* = 35)	None	31 (93.9%)	35 (100.0%)	0.232
	Condom	2 (6.1%)	0 (0.0%)	
Smoking status*** (*n* = 33/*n* = 35)	No	32 (97.0%)	33 (94.3%)	0.590
	Yes	1 (3.0%)	2 (5.7%)	
Alcohol use*** (*n* = 34/*n* = 35)	No	34 (100.0%)	35 (100.0%)	NA
Chronic disease*** (*n* = 34/*n* = 35)	No	5 (14.7%)	12 (34.3%)	0.059
	Yes	29 (85.3%)	23 (65.7%)	

NA; Non Applicable, * Normally distributed data presented as mean ± standard deviation; compared by independent samples *t*-test.** Non-normally distributed data presented as median (minimum–maximum); compared by Mann–Whitney U test. *** Categorical variables presented as n (%); compared using the Chi-square or Fisher’s exact test, as appropriate.

Grade 1, 2, and 3 tumors were found in 6.1, 87.9, and 6.1% of the brachytherapy group, and in 62.9, 34.3, and 2.9% of the surgery group, respectively (*p* < 0.001). Stage IA disease was more frequent in the surgery group (97.1% vs. 73.5%), whereas stages IB–II were more common in the brachytherapy group (*p* = 0.005). Laparotomy was performed in 51.9% vs. 14.3%, laparoscopy in 44.4% vs. 82.9%, and robotic surgery in 3.7% vs. 2.9% (*p* = 0.002). Pelvic lymphadenectomy was done in 88.9% vs. 100% (*p* = 0.043), paraaortic in 55.6% vs. 17.1% (*p* = 0.002), and sentinel node dissection in 14.8% vs. 20.0% (*p* = 0.742) (Table 2).

**Table 2:** Pathological grade, stage, and lymph node dissection by treatment group.

Variable		Brachytherapy	Surgery	*p*-value
Tumor grade*** (*n* = 33/*n* = 35)	Grade 1	2_a_ (6.1%)	22_b_ (62.9%)	**<0.001**
	Grade 2	29_a_ (87.9%)	12_b_ (34.3%)	
	Grade 3	2_a_ (6.1%)	1_a_ (2.9%)	
Tumor stage*** (*n* = 34/*n* = 35)	Stage 1A	25_a_ (73.5%)	34_b_ (97.1%)	**0.005**
	Stage 1B	6_a_ (17.6%)	0_b_ (0.0%)	
	Stage 2	3_a_ (8.8%)	1_a_ (2.9%)	
Surgical method*** (*n* = 27/*n* = 35)	Laparotomy	14_a_ (51.9%)	5_b_ (14.3%)	**0.002**
	Laparoscopy	12_a_ (44.4%)	29_b_ (82.9%)	
	Robotic	1_a_ (3.7%)	1_a_ (2.9%)	
Pelvic LND*** (*n* = 27/*n* = 35)	Yes	24_a_ (88.9%)	35_b_ (100.0%)	**0.043**
	No	3_a_ (11.1%)	0_b_ (0.0%)	
Paraaortic LND*** (*n* = 27/*n* = 35)	Yes	15_a_ (55.6%)	6_b_ (17.1%)	**0.002**
	No	12_a_ (44.4%)	29_b_ (82.9%)	
Sentinel LND*** (*n* = 27/*n* = 35)	Yes	4 (14.8%)	7 (20.0%)	0.742
	No	23 (85.2%)	28 (80.0%)	

LND, Lymph Node Dissection; NS, Not Significant (*p* > 0.05). ***Categorical variables presented as n (%); compared using the Chi-square or Fisher’s exact test, as appropriate. Categories with the same subscript letter are not significantly different in terms of column proportions (*p* > 0.05). Bold values indicate a statistically significant difference (*p* > 0.05).

### Sexual function outcomes (FSFI and NSSS index scores and subdomains)

Sexual function and pain assessments are presented in Table 3. The median Female Sexual Function Index (FSFI) score was 2 (2–20.3) versus 2 (2–19.6) in the brachytherapy and surgery groups, respectively (*p* = 0.459). Male sexual satisfaction scores were 20 (20–83) versus 20 (20–90) (*p* = 0.492). Sexual desire scores were 1.2 (1,2–3,6) versus 1.2 (0–3.0) (*p* = 0.442), arousal scores were 0 (0–3.9) versus 0 (0–3.9) (*p* = 0.881), lubrication scores were 0 (0–3.9) versus 0 (0–4.2) (*p* = 0.234), orgasm scores were 0 (0–4.0) versus 0 (0–3.2) (*p* = 0.336), satisfaction scores were 0.8 (0.8–4.4) versus 0.8 (0.8–3.6) (*p* = 0.513), and pain scores were 0 (0–6) versus 0 (0–6) (*p* = 0.310) (Table 3).

**Table 3:** Sexual function scores and pain assessment by treatment group.

Variable	Brachytherapy	Surgery	*p*-value
Female FSFI score** (*n* = 32/*n* = 32)	2 (2–20.3)	2 (2–19.6)	0.459
Male sexual satisfaction** (*n* = 25/*n* = 28)	20 (20–83)	20 (20–90)	0.492
Sexual desire** (*n* = 31/*n* = 32)	1.2 (1.2–3.6)	1,2 (0–3)	0.442
Arousal** (*n* = 31/*n* = 32)	0 (0–3.9)	0 (0–3.9)	0.881
Lubrication** (*n* = 31/*n* = 32)	0 (0–3.9)	0 (0–4.2)	0.234
Orgasm** (*n* = 31/*n* = 32)	0 (0–4)	0 (0–3.2)	0.336
Satisfaction** (*n* = 31/*n* = 32)	0.8 (0.8–4.4)	0.8 (0.8–3.6)	0.513
Pain** (*n* = 31/*n* = 32)	0 (0–6)	0 (0–6)	0.310

FSFI, Female Sexual Function Index; ** Non-normally distributed data presented as median (minimum–maximum); compared by Mann–Whitney U test.

**Figure 2:**
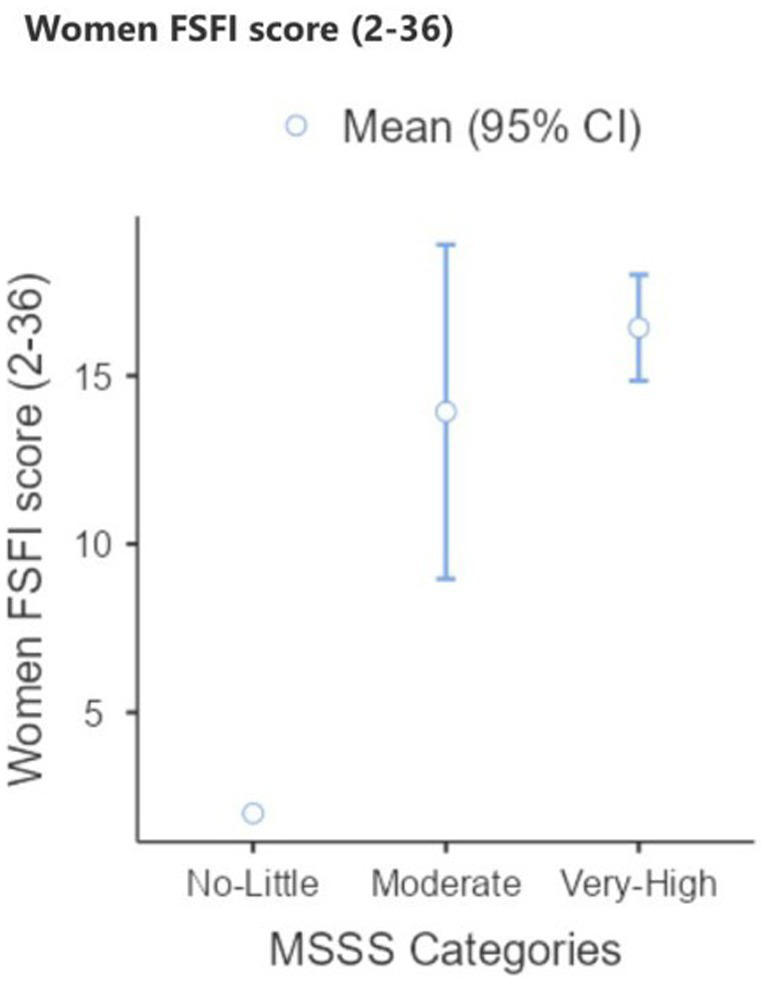
Women FSFI score (2–36).

Sexual function scores obtained from the FSFI and NSSS were compared between the brachytherapy and surgery groups. Their distributions are presented using box plots (Figure 1). Additionally, education level was recorded as a key sociodemographic factor. The distribution of education levels by treatment group is shown in Figure 2, indicating that the majority of patients in both groups were primary school graduates, with no university graduates in the brachytherapy group.

**Figure 3:**
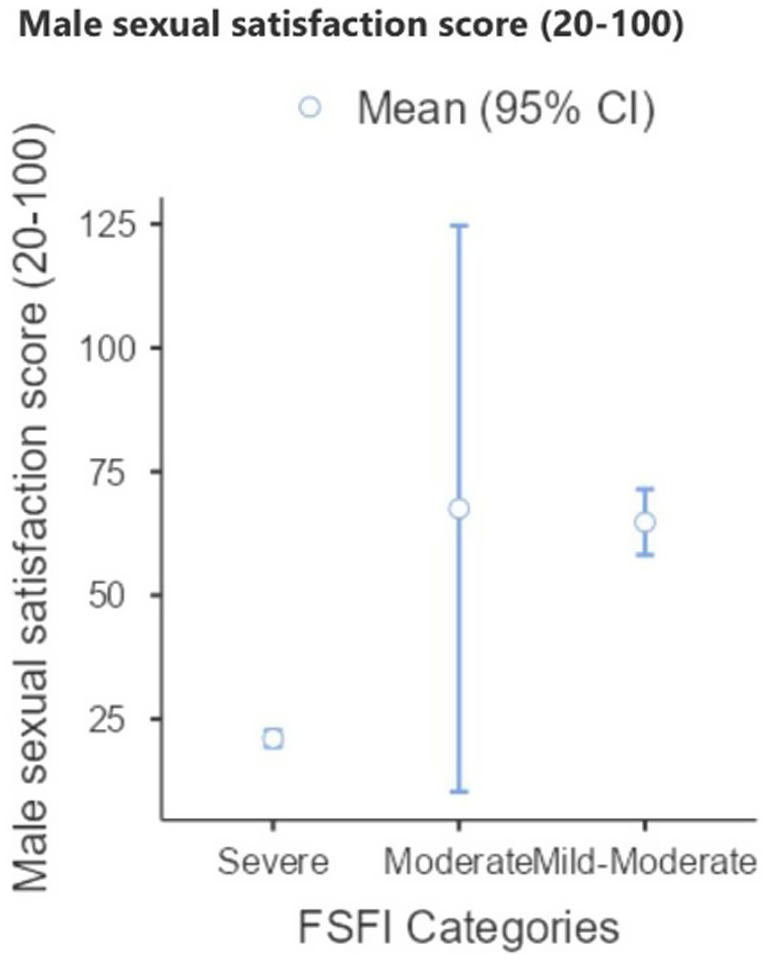
Male satisfaction score (2–100).

**Figure 4:**
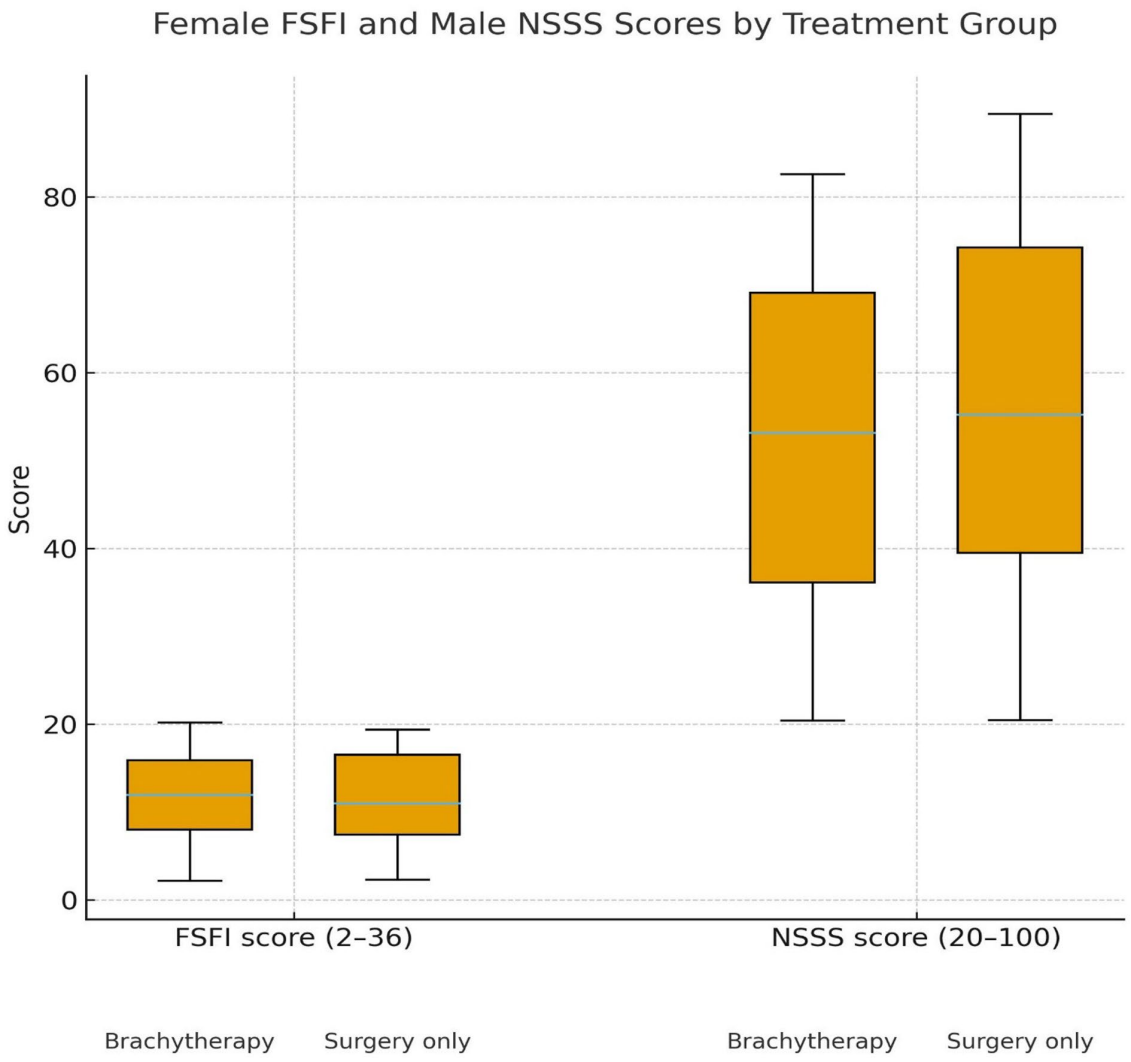
FSFI and NSSS scores in brachytherapy and surgery groups.

After controlling for age, no significant differences were found between the surgery-only and surgery plus brachytherapy groups in either female sexual function (FSFI) or male sexual satisfaction (NSSS) (Table 4). Although FSFI scores were slightly lower in the brachytherapy group, the difference was not significant (*p* > 0.05). Women whose partners reported higher satisfaction had higher mean FSFI scores, but this trend was not statistically significant (Figure 2). Similarly, male satisfaction increased progressively with higher female FSFI categories (Figure 3). Box plots demonstrated comparable FSFI and NSSS distributions between groups, with median scores similarly low in both (Figure 4). Overall, sexual function and satisfaction were mainly influenced by age rather than treatment modality.

**Table 4:** ANCOVA results for female sexual function and male sexual satisfaction scores adjusted for age.

Dependent variable	Source	Sum of squares	df	Mean square	F	*p*-value
Female FSFI score (2–36)	Group	7.49	1	7.49	0.21	0.651
	Age	912.77	1	912.77	25.26	< 0.001
	Residuals	2204.24	61	36.14		
Male sexual satisfaction score (20–100)	Group	7.79	1	7.79	0.02	0.886
	Age	8775.88	1	8775.88	23.55	< 0.001
	Residuals	18629.36	50	372.59		

Dependent variable	Contrast (surgery—brachytherapy)	Estimate	SE	t	*p*-value
Female FSFI score	−0.71	1.55	−0.46	0.651	
Male sexual satisfaction score	0.78	5.42	0.14	0.886	

FSFI, Female Sexual Function Index; ANCOVA, Analysis of Covariance; SE, Standard Error. ANCOVA analyses were adjusted for age as a covariate. No significant differences were observed between groups after adjustment, while age remained a significant predictor for both female and male sexual outcomes (*p* < 0.001).

## Discussion

This study is the first to examine both male and female sexual function specifically in the context of endometrial cancer. We analyzed the demographic characteristics of patients diagnosed with endometrial cancer and compared sexual function in both the patients and their partners across two treatment groups: those who underwent surgery alone and those who received surgery with additional brachytherapy. To our knowledge, this is the first study to comprehensively assess sexual function in both partners following treatment for endometrial cancer.

Our research differs from previous studies, including the most comparable work by Nowesieski et al. (14), which evaluated sexual function in the broader context of gynecologic cancers. In contrast, our study focuses exclusively on endometrial cancer and includes a larger, more targeted sample of 69 patient-partner pairs. Additionally, it uniquely evaluates sexual function in both female patients and their partners following either surgery alone or adjuvant brachytherapy. In this regard, our study provides a more comprehensive and focused assessment across a broader range of parameters and patient profiles.

In our cohort, the brachytherapy group consisted of 34 patients, while 35 received only surgical treatment. When examining demographic characteristics, we found that the majority of participants in both groups were primary school graduates. This finding contrasts with the study by Aygın and Eti Aslan, which involved the Turkish adaptation of the Female Sexual Function Index (FSFI) and reported that most participants were employed and had completed secondary education (15). Despite these differences in educational background, both studies yielded similar findings in terms of parity, with most women having two or three children. This suggests that parity may be a more consistent demographic variable across different populations studied for sexual function, while education level and employment status may vary depending on the clinical setting and population characteristics.

Tugut et al. (16) examined the validity and reliability of the New Sexual Satisfaction Scale in a sample of adults in Turkey. The study found that the two-factor structure of the scale was preserved in the Turkish adult sample and that it demonstrated a high level of reliability. However, the majority of participants consisted of unemployed women with primary school education and employed men with university degrees (16). In our study, while most participants were similarly primary school graduates, the distribution of educational status by gender was not clearly distinguished. Variables such as education and employment status need to be examined in more detail regarding their impact on sexual satisfaction.

Our study investigated both female and male sexual function in endometrial cancer patients who underwent either surgery alone or additional brachytherapy. Despite slight demographic differences between the group such as age, parity, and educational status, our results revealed no statistically significant differences in overall sexual function or pain scores between treatment modalities. The median Female Sexual Function Index (FSFI) scores were equally low in both groups, suggesting that sexual dysfunction is common regardless of the addition of brachytherapy. Similarly, male partners reported comparable levels of sexual satisfaction in both groups, indicating that the presence of brachytherapy did not significantly influence partner sexual experience.

In our study, pelvic lymph node dissection was performed in the majority of patients in both groups (88.9% vs. 100.0%; *p* = 0.043), while paraaortic lymph node dissection was significantly more frequent in the brachytherapy group (55.6% vs. 17.1%; *p* = 0.002). Although no significant difference was observed in overall FSFI or NSSS scores between groups, the higher rate of paraaortic dissection in the brachytherapy group may partially contribute to the more pronounced sexual dysfunction observed in this cohort. This aligns with findings by Fujii (17) who demonstrated that extensive lymphadenectomy, particularly involving paraaortic regions, may lead to autonomic nerve injury, thereby negatively impacting bladder, bowel, and sexual function in gynecologic oncology patients. While our study did not specifically assess nerve preservation, the higher incidence of sexual dysfunction in patients undergoing brachytherapy, combined with more extensive lymph node dissection, suggests a possible cumulative effect on pelvic autonomic innervation. These findings support the need for further research into nerve-sparing surgical approaches, particularly in patients likely to require adjuvant radiotherapy.

In contrast, the study by Nowosielski et al. (14), which included 30 gynecologic cancer survivors and their male partners, found more nuanced gender differences in sexual adaptation following treatment. Specifically, while female survivors reported a decline in sexual frequency, male partners paradoxically reported an increase Moreover, women in that study demonstrated higher sexual inhibitory tone and more body image concerns than their partners, revealing a potential divergence in sexual recovery trajectories within couples after cancer treatment (14).

Unlike the findings of Nowosielski et al., where inter-partner discrepancies in sexual interest and perception were clearly documented, our study observed no significant difference in sexual function between genders or between treatment arms. This could be due to differences in sample characteristics, cancer types, or cultural factors, including conservative attitudes toward sexuality, as the majority of our participants were primary school graduates and did not use contraceptives, indicating a potentially lower baseline level of sexual activity and openness. Additionally, in our study population, none of the patients reported alcohol use and over 95% were nonsmokers, which may also reflect more traditional lifestyle patterns and potentially contribute to sexual conservatism.

In our study, female sexual dysfunction was prevalent in both the brachytherapy and surgery-only groups, with equally low median FSFI scores. This aligns with the findings of Damast et al., who reported sexual dysfunction in 81% of endometrial cancer survivors treated with high-dose-rate intravaginal brachytherapy, with impaired scores across all FSFI domains (18). Notably, our data suggest that the addition of brachytherapy did not further worsen sexual outcomes, possibly due to already low baseline sexual activity and sociocultural factors, such as limited education and conservative sexual behavior. While Damast et al. identified laparotomy as a predictor of poorer sexual function, in our cohort, the higher laparotomy rate in the brachytherapy group did not translate into statistically worse FSFI scores, highlighting the complex interplay of treatment and demographic influences on sexual health.

These findings underscore the importance of contextualizing sexual health outcomes in oncology not only by treatment modality but also by sociocultural background, gender dynamics, and relationship factors.

In a study reported by Datta et al. (19) which included 132 patients who underwent surgery alone, surgery followed by brachytherapy, or surgery followed by chemoradiotherapy, 89% of participants continued to report low sexual function scores 1 year after treatment. Being over the age of 50 and having an education level below a university degree were identified as significant predictors of impaired sexual function. The study also emphasized that women who underwent surgery alone reported better quality of life compared to those who received adjuvant therapy, with the lowest quality of life scores observed in women who received chemoradiotherapy (19). In contrast, in our study, sexual function remained similarly low across all treatment groups, with no statistically significant differences found between them. This suggests that factors beyond treatment modality such as age, partner relationship quality, communication levels, and psychological adaptation may play a more critical role in determining post-treatment sexual health outcomes.

Our results align partially with a recent study comparing early-stage endometrial cancer patients who received intravaginal brachytherapy (IVB) with those treated by surgery alone, which reported similar outcomes in terms of quality of life and female sexual function, although male partner function was not evaluated (20). In contrast, another study focusing on high-dose-rate (HDR) IVB involving treatment of a ≥ 6 cm vaginal cuff found significantly higher rates of sexual dysfunction among brachytherapy patients compared to those who had surgery alone (21). The discrepancy may stem from differences in radiation dose, vaginal length irradiated, and inclusion criteria. Notably, our cohort had relatively short vaginal cuff targets, and sociocultural characteristics such as low educational attainment and conservative sexual practices may have contributed to universally low FSFI scores, thus diminishing the observable difference between treatment arms.

Emerging technologies particularly artificial intelligence (AI) enabled decision support are rapidly reshaping early diagnosis and personalized oncologic care, with potential downstream effects on survivorship domains such as sexual function. Recent overviews emphasize that AI can integrate heterogeneous inputs (clinical, imaging, pathology, molecular) to enhance risk stratification, predict treatment response, and standardize complex decisions across centers. In endometrial cancer, such platforms could refine indications for adjuvant radiotherapy and tailor brachytherapy dose/volume parameters, potentially minimizing vaginal toxicity and pelvic autonomic sequelae that contribute to female sexual dysfunction. Beyond planning, AI-driven symptom triage and digital counseling tools may also support timely identification of dyspareunia, lubrication issues, and psychosexual distress, facilitating earlier referral to pelvic floor rehabilitation and couple-based interventions (22).

Converging evidence further suggests that AI-based clinical decision support systems (CDSS) can operationalize “learning health system” principles continuous model updating, calibration checks, and deployment monitoring to deliver patient-specific recommendations while flagging uncertainty (23). Applied to our context, CDSS could estimate individual toxicity probabilities (e.g., post-IVB vaginal stenosis risk) from preoperative and dosimetric features; surface modifiable risk factors (e.g., cuff length, dose to OARs) for shared decision-making; and trigger structured follow-up for couples with predicted high risk of sexual dysfunction. Notwithstanding these opportunities, both reviews stress critical safeguards: robust multicenter datasets; bias auditing across age/education/cultural strata; transparent model reporting; and prospective external validation before clinical adoption (21). Our findings of uniformly low FSFI/NSSS scores likely shaped by sociocultural context underscore that future AI/CDSS must be evaluated not only for diagnostic/therapeutic accuracy but also for equity and real-world impact on couple-centered sexual health outcomes.

This study has several limitations. The study population was characterized by low educational attainment, limited contraceptive use, and conservative sexual behavior, which may have led to universally low FSFI scores and potentially masked more subtle treatment-related effects as well as the number of patients included to the study. Sexual function was assessed at a single time point post-treatment, precluding evaluation of longitudinal changes. Additionally, validated sexual function questionnaires for male partners were not employed; instead, partner sexual satisfaction was self-reported in a simplified manner. Finally, psychological factors, hormonal status, and relationship quality which are known to influence sexual function were not systematically evaluated.

Age-related genitourinary syndrome, exclusion of chronic comorbidities, and the relatively small sample size limit generalizability. Preoperative sexual function could not be reliably assessed, preventing baseline comparisons. Despite these limitations, the inclusion of male partners provides valuable couple-based insights, supporting future larger multicenter studies.

## Conclusion

Brachytherapy did not independently affect female sexual function or male partner satisfaction. Sexual outcomes were mainly age-related, and strong correlations between female dysfunction and partner satisfaction highlight the need for couple-focused survivorship care.

## Data Availability

The raw data supporting the conclusions of this article will be made available by the authors, without undue reservation.
